# *N*-(3,4-Dichloro­phen­yl)maleamic acid

**DOI:** 10.1107/S160053681002129X

**Published:** 2010-06-16

**Authors:** B. Thimme Gowda, Miroslav Tokarčík, K. Shakuntala, Jozef Kožíšek, Hartmut Fuess

**Affiliations:** a Department of Chemistry, Mangalore University, Mangalagangotri 574 199, Mangalore, India; b Faculty of Chemical and Food Technology, Slovak Technical University, Radlinského 9, SK-812 37 Bratislava, Slovak Republic; c Institute of Materials Science, Darmstadt University of Technology, Petersenstrasse 23, D-64287 Darmstadt, Germany

## Abstract

The asymmetric unit of the title compound, C_10_H_7_Cl_2_NO_3_, contains two unique mol­ecules, both being stabilized by an intra­molecular O—H⋯O hydrogen bond within their maleamic units. In the crystal structure, inter­molecular N—H⋯O hydrogen bonds link the mol­ecules into chains extending along [1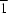 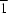] which are further assembled into sheets *via* short inter­molecular C—Cl⋯O=C contacts [3.102 (2) and 3.044 (2) Å].

## Related literature

For studies on the effect of ring- and side-chain substitutions on the crystal structures of amides, see: Gowda *et al.* (2009 ▶, 2010 ▶); Lo & Ng (2009 ▶); Prasad *et al.* (2002 ▶); Shakuntala *et al.* (2009 ▶). For short halogen–oxygen contacts, see: Fourmigué (2009 ▶); Legon (1999 ▶).
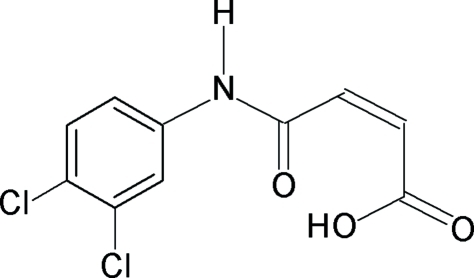


## Experimental

### 

#### Crystal data

C_10_H_7_Cl_2_NO_3_
*M* *_r_* = 260.07
Triclinic, 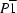

*a* = 7.1959 (7) Å
*b* = 11.6234 (10) Å
*c* = 13.1399 (14) Å
α = 85.116 (8)°
β = 75.060 (9)°
γ = 81.205 (7)°
*V* = 1048.19 (18) Å^3^
*Z* = 4
Mo *K*α radiation
μ = 0.61 mm^−1^
*T* = 295 K
0.54 × 0.28 × 0.11 mm

#### Data collection

Oxford Diffraction Gemini R, CCD diffractometer
Absorption correction: multi-scan (*CrysAlis PRO* RED; Oxford Diffraction, 2009 ▶) *T*_min_ = 0.870, *T*_max_ = 0.969
11933 measured reflections
3897 independent reflections
3075 reflections with *I* > 2σ(*I*)
*R*_int_ = 0.026

#### Refinement

*R*[*F* ^2^ > 2σ(*F* ^2^)] = 0.033
*wR*(*F* ^2^) = 0.083
*S* = 1.03
3897 reflections
295 parameters
2 restraints
H atoms treated by a mixture of independent and constrained refinement
Δρ_max_ = 0.33 e Å^−3^
Δρ_min_ = −0.23 e Å^−3^

### 

Data collection: *CrysAlis PRO CCD* (Oxford Diffraction, 2009 ▶); cell refinement: *CrysAlis PRO CCD*; data reduction: *CrysAlis PRO RED* (Oxford Diffraction, 2009 ▶); program(s) used to solve structure: *SHELXS97* (Sheldrick, 2008 ▶); program(s) used to refine structure: *SHELXL97* (Sheldrick, 2008 ▶); molecular graphics: *ORTEP-3* (Farrugia, 1997 ▶) and *DIAMOND* (Brandenburg, 2002 ▶); software used to prepare material for publication: *SHELXL97*, *PLATON* (Spek, 2009 ▶) and *WinGX* (Farrugia, 1999 ▶).

## Supplementary Material

Crystal structure: contains datablocks I, global. DOI: 10.1107/S160053681002129X/xu2768sup1.cif

Structure factors: contains datablocks I. DOI: 10.1107/S160053681002129X/xu2768Isup2.hkl

Additional supplementary materials:  crystallographic information; 3D view; checkCIF report

## Figures and Tables

**Table 1:** Hydrogen-bond geometry (Å, °)

*D*—H⋯*A*	*D*—H	H⋯*A*	*D*⋯*A*	*D*—H⋯*A*
N1—H1*N*⋯O6^i^	0.86	2.03	2.869 (2)	165
N2—H2*N*⋯O3^ii^	0.86	2.03	2.873 (2)	166
O2—H2*A*⋯O1	0.89 (2)	1.61 (2)	2.496 (2)	171 (3)
O5—H5*A*⋯O4	0.90 (2)	1.59 (2)	2.492 (2)	174 (3)

Symmetry codes: (i) 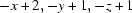; (ii) 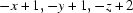.

**Fig. 1.**
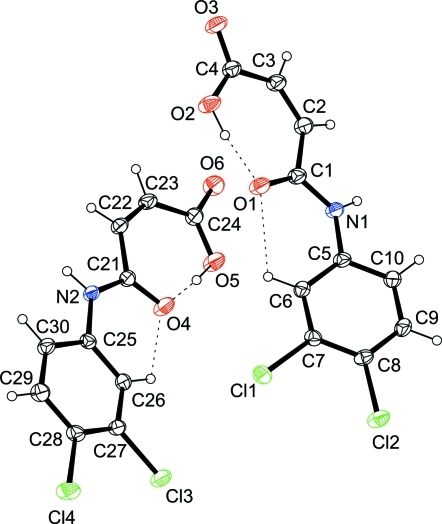
Molecular structure of (I) showing the atom labelling scheme. Displacement ellipsoids are drawn at the 30% probability level. H atoms are represented as small spheres of arbitrary radii and short intramolecular O—H···O bonds as dashed lines.

**Fig. 2.**
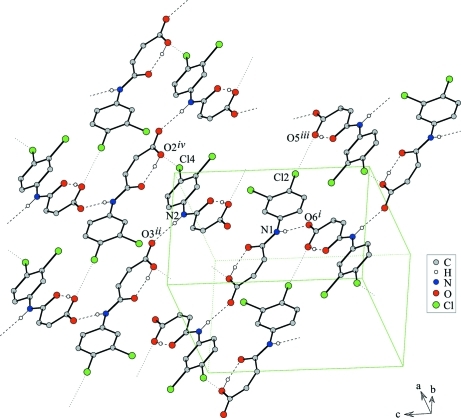
Part of the crystal structure of (I) showing the two-dimensional networks of molecules linked by N–H···O hydrogen bonds and short Cl···O contacts. Symmetry codes (i): -x + 2, -y + 1, -z + 1; (ii) -x + 1, -y + 1, -z + 2; (iii) -x + 2, -y + 2, -z + 1; (iv) -x + 1, -y + 2, -z + 2. H atoms not involved in hydrogen bonding were omitted. Hydrogen bonds are shown as dashed lines, Cl···O contacts as dotted lines.

## supplementary crystallographic information

### Comment

In the present work, as a part of studying the effect of ring and side chain substitutions on the crystal structures of biologically significant amides (Gowda *et al.*, 2009, 2010; Shakuntala *et al.*, 2009; Prasad *et al.*, 2002), the crystal structure of *N*-(3,4-dichlorophenyl)maleamic acid (I) has been determined (Fig. 1).

The asymmetric unit of the cell contains two molecules. In the first molecule, which significantly deviates from planarity, the torsion angle C6—C5—N1—C1 = 24.9 (3)° defines the orientation of the phenyl ring towards the central amide group —NHCO—. The atoms of maleamic acid moiety do not fit very well to a plane (r.m.s. deviation = 0.077\%A). It makes a dihedral angle of 27.5 (1)° with the phenyl ring. The geometry of the second molecule is almost planar as shown by the small dihedral angle of 1.9 (1)° formed by the planes of phenyl ring and maleamic acid moiety. Each maleamic acid moiety includes a short intramolecular hydrogen bond O—H···O (Table 1). The bond lengths C2–C3 = 1.336 (3) and C22–C23 = 1.333 (3)\%A clearly indicate the double bond character.

In the crystal structure (Fig. 2), the intermolecular N–H···O hydrogen bonds link the molecules into chains extending along the [1 - 1 -1]direction. These chains are further assembled by short Cl···O contacts of the length 3.102 (2) and 3.044 (2)Å to form the sheet like structure.

Our data for the C–Cl···O halogen bonds are in agreement with the observations of others (Fourmigué, 2009, Legon, 1999).

### Experimental

The solution of maleic anhydride (0.025 mol) in toluene (25 ml) was treated dropwise with the solution of 3,4-dichloroaniline (0.025 mol) also in toluene (20 ml) with constant stirring. The resulting mixture was warmed with stirring for over 30 min and set aside for an additional 30 min at room temperature for completion of the reaction. The mixture was then treated with dilute hydrochloric acid to remove the unreacted 3,4-dichloroaniline. The resultant solid *N*-(3,4-dichlorophenyl)maleamic acid was filtered under suction and washed thoroughly with water to remove the unreacted maleic anhydride and maleic acid. It was recrystallized to constant melting point from ethanol. The purity of the compound was checked by elemental analysis and characterized by its infrared spectra.

Block like colourless single crystals used in X-ray diffraction studies were grown in an ethanol solution by slow evaporation at room temperature.

### Refinement

H atoms bonded to C and N atoms were positioned with idealized geometry (C—H = 0.93 Å, N—H = 0.86 Å) and refined using a riding model. H atoms of carboxyl groups were visible in difference maps and were refined freely with O—H distances restrained to 0.90 (3) Å. The *U*_iso_(H) values were set at 1.2*U*_eq_(C aromatic, N) and 1.5*U*_eq_(O).

### Crystal data

**Table:**

C_10_H_7_Cl_2_NO_3_	*Z* = 4
*M**_r_* = 260.07	*F*(000) = 528
Triclinic, *P*1	*D*_x_ = 1.648 Mg m^−^^3^
Hall symbol: -P 1	Mo *K*α radiation, λ = 0.71073 Å
*a* = 7.1959 (7) Å	Cell parameters from 5934 reflections
*b* = 11.6234 (10) Å	θ = 1.6–28.1°
*c* = 13.1399 (14) Å	µ = 0.61 mm^−^^1^
α = 85.116 (8)°	*T* = 295 K
β = 75.060 (9)°	Block, colourless
γ = 81.205 (7)°	0.54 × 0.28 × 0.11 mm
*V* = 1048.19 (18) Å^3^	

### Data collection

**Table:**

Oxford Diffraction Gemini R, CCD diffractometer	3897 independent reflections
graphite	3075 reflections with *I* > 2σ(*I*)
Detector resolution: 10.434 pixels mm^-1^	*R*_int_ = 0.026
ω scans	θ_max_ = 25.5°, θ_min_ = 1.8°
Absorption correction: multi-scan (*CrysAlis PRO* RED; Oxford Diffraction, 2009)	*h* = −8→8
*T*_min_ = 0.870, *T*_max_ = 0.969	*k* = −14→14
11933 measured reflections	*l* = −15→15

### Refinement

**Table:**

Refinement on *F*^2^	Primary atom site location: structure-invariant direct methods
Least-squares matrix: full	Secondary atom site location: difference Fourier map
*R*[*F*^2^ > 2σ(*F*^2^)] = 0.033	Hydrogen site location: inferred from neighbouring sites
*wR*(*F*^2^) = 0.083	H atoms treated by a mixture of independent and constrained refinement
*S* = 1.03	*w* = 1/[σ^2^(*F*_o_^2^) + (0.0379*P*)^2^ + 0.3773*P*] where *P* = (*F*_o_^2^ + 2*F*_c_^2^)/3
3897 reflections	(Δ/σ)_max_ = 0.001
295 parameters	Δρ_max_ = 0.33 e Å^−^^3^
2 restraints	Δρ_min_ = −0.23 e Å^−^^3^

### Special details

**Table:**

Geometry. All e.s.d.'s (except the e.s.d. in the dihedral angle between two l.s. planes) are estimated using the full covariance matrix. The cell e.s.d.'s are taken into account individually in the estimation of e.s.d.'s in distances, angles and torsion angles; correlations between e.s.d.'s in cell parameters are only used when they are defined by crystal symmetry. An approximate (isotropic) treatment of cell e.s.d.'s is used for estimating e.s.d.'s involving l.s. planes.
Refinement. Refinement of *F*^2^ against ALL reflections. The weighted *R*-factor *wR* and goodness of fit *S* are based on *F*^2^, conventional *R*-factors *R* are based on *F*, with *F* set to zero for negative *F*^2^. The threshold expression of *F*^2^ > σ(*F*^2^) is used only for calculating *R*-factors(gt) *etc*. and is not relevant to the choice of reflections for refinement. *R*-factors based on *F*^2^ are statistically about twice as large as those based on *F*, and *R*- factors based on ALL data will be even larger.

### Fractional atomic coordinates and isotropic or equivalent isotropic displacement parameters (Å^2^)

**Table:**

	*x*	*y*	*z*	*U*_iso_*/*U*_eq_	
C1	0.6769 (3)	0.57748 (17)	0.60126 (16)	0.0368 (5)	
C2	0.6821 (3)	0.45235 (17)	0.58531 (16)	0.0408 (5)	
H2	0.7495	0.4269	0.519	0.049*	
C3	0.6022 (3)	0.37134 (17)	0.65399 (16)	0.0416 (5)	
H3	0.6257	0.2973	0.6277	0.05*	
C4	0.4826 (3)	0.37742 (17)	0.76479 (16)	0.0386 (5)	
C5	0.7512 (3)	0.76165 (16)	0.50046 (15)	0.0327 (4)	
C6	0.7421 (3)	0.83188 (16)	0.58292 (15)	0.0330 (4)	
H6	0.7353	0.7995	0.6508	0.04*	
C7	0.7433 (3)	0.95030 (16)	0.56238 (15)	0.0313 (4)	
C8	0.7540 (3)	1.00008 (16)	0.46162 (16)	0.0329 (4)	
C9	0.7642 (3)	0.92903 (18)	0.38049 (16)	0.0389 (5)	
H9	0.7725	0.9614	0.3125	0.047*	
C10	0.7623 (3)	0.81111 (18)	0.39948 (16)	0.0380 (5)	
H10	0.7684	0.7642	0.3445	0.046*	
N1	0.7501 (2)	0.63960 (14)	0.51337 (13)	0.0378 (4)	
H1N	0.8028	0.6006	0.458	0.045*	
O1	0.6107 (2)	0.62179 (12)	0.68815 (12)	0.0503 (4)	
O2	0.4523 (3)	0.47244 (13)	0.81526 (12)	0.0562 (5)	
H2A	0.506 (4)	0.530 (2)	0.776 (2)	0.084*	
O3	0.4146 (2)	0.29115 (13)	0.80735 (12)	0.0528 (4)	
Cl1	0.73228 (8)	1.03670 (4)	0.66563 (4)	0.04481 (16)	
Cl2	0.75788 (8)	1.14770 (4)	0.43458 (4)	0.04553 (16)	
C21	0.8620 (3)	0.74292 (16)	0.90501 (15)	0.0325 (4)	
C22	0.8539 (3)	0.61620 (16)	0.92152 (16)	0.0357 (5)	
H22	0.7904	0.5909	0.9888	0.043*	
C23	0.9263 (3)	0.53360 (17)	0.85204 (16)	0.0386 (5)	
H23	0.9045	0.459	0.8795	0.046*	
C24	1.0349 (3)	0.53723 (17)	0.73945 (16)	0.0375 (5)	
C25	0.7588 (3)	0.92609 (15)	1.00107 (15)	0.0286 (4)	
C26	0.8324 (3)	1.00323 (16)	0.91955 (15)	0.0309 (4)	
H26	0.9001	0.9761	0.8538	0.037*	
C27	0.8038 (3)	1.12116 (16)	0.93712 (15)	0.0314 (4)	
C28	0.7036 (3)	1.16303 (16)	1.03496 (16)	0.0335 (4)	
C29	0.6323 (3)	1.08583 (17)	1.11585 (16)	0.0383 (5)	
H29	0.566	1.1131	1.1817	0.046*	
C30	0.6590 (3)	0.96819 (17)	1.09950 (15)	0.0350 (5)	
H30	0.6101	0.9165	1.1544	0.042*	
N2	0.7781 (2)	0.80432 (13)	0.99092 (12)	0.0317 (4)	
H2N	0.7298	0.7645	1.0471	0.038*	
O4	0.9388 (3)	0.78924 (12)	0.81921 (11)	0.0524 (4)	
O5	1.0862 (3)	0.63417 (13)	0.69133 (12)	0.0544 (5)	
H5A	1.039 (4)	0.693 (2)	0.735 (2)	0.082*	
O6	1.0751 (3)	0.44786 (13)	0.69212 (12)	0.0558 (4)	
Cl3	0.89907 (9)	1.21571 (4)	0.83501 (4)	0.04603 (16)	
Cl4	0.66462 (9)	1.31041 (4)	1.05637 (5)	0.05015 (17)	

### Atomic displacement parameters (Å^2^)

**Table:**

	*U*^11^	*U*^22^	*U*^33^	*U*^12^	*U*^13^	*U*^23^
C1	0.0428 (12)	0.0320 (10)	0.0322 (11)	−0.0102 (9)	0.0005 (9)	−0.0035 (9)
C2	0.0538 (13)	0.0333 (11)	0.0300 (11)	−0.0105 (10)	0.0036 (10)	−0.0078 (9)
C3	0.0575 (14)	0.0274 (10)	0.0353 (12)	−0.0093 (9)	0.0006 (10)	−0.0069 (9)
C4	0.0474 (13)	0.0307 (11)	0.0344 (11)	−0.0083 (9)	−0.0027 (10)	−0.0021 (9)
C5	0.0353 (11)	0.0302 (10)	0.0297 (11)	−0.0107 (8)	0.0011 (8)	−0.0020 (8)
C6	0.0381 (11)	0.0334 (10)	0.0262 (10)	−0.0089 (9)	−0.0032 (9)	−0.0009 (8)
C7	0.0317 (10)	0.0309 (10)	0.0296 (11)	−0.0059 (8)	−0.0019 (8)	−0.0075 (8)
C8	0.0291 (10)	0.0296 (10)	0.0349 (11)	−0.0060 (8)	0.0017 (8)	0.0001 (8)
C9	0.0467 (13)	0.0407 (12)	0.0258 (10)	−0.0109 (10)	−0.0008 (9)	0.0012 (9)
C10	0.0472 (13)	0.0381 (11)	0.0267 (11)	−0.0140 (9)	0.0001 (9)	−0.0049 (9)
N1	0.0530 (11)	0.0285 (9)	0.0271 (9)	−0.0117 (8)	0.0035 (8)	−0.0061 (7)
O1	0.0782 (11)	0.0318 (8)	0.0324 (8)	−0.0177 (7)	0.0094 (8)	−0.0081 (6)
O2	0.0877 (13)	0.0356 (9)	0.0338 (9)	−0.0224 (8)	0.0155 (8)	−0.0073 (7)
O3	0.0753 (11)	0.0364 (8)	0.0393 (9)	−0.0224 (8)	0.0059 (8)	0.0026 (7)
Cl1	0.0622 (4)	0.0363 (3)	0.0360 (3)	−0.0100 (2)	−0.0076 (3)	−0.0109 (2)
Cl2	0.0530 (3)	0.0293 (3)	0.0473 (3)	−0.0066 (2)	−0.0007 (3)	0.0026 (2)
C21	0.0427 (12)	0.0258 (10)	0.0268 (10)	−0.0041 (8)	−0.0048 (9)	−0.0029 (8)
C22	0.0488 (13)	0.0272 (10)	0.0272 (10)	−0.0082 (9)	−0.0009 (9)	−0.0010 (8)
C23	0.0544 (13)	0.0228 (10)	0.0348 (11)	−0.0071 (9)	−0.0032 (10)	−0.0022 (8)
C24	0.0469 (13)	0.0291 (11)	0.0329 (11)	−0.0018 (9)	−0.0044 (9)	−0.0052 (9)
C25	0.0314 (10)	0.0249 (9)	0.0295 (10)	−0.0034 (8)	−0.0065 (8)	−0.0053 (8)
C26	0.0367 (11)	0.0288 (10)	0.0252 (10)	−0.0041 (8)	−0.0031 (8)	−0.0059 (8)
C27	0.0339 (11)	0.0269 (10)	0.0319 (11)	−0.0072 (8)	−0.0043 (9)	0.0002 (8)
C28	0.0377 (11)	0.0246 (9)	0.0358 (11)	−0.0019 (8)	−0.0041 (9)	−0.0091 (8)
C29	0.0429 (12)	0.0335 (11)	0.0326 (11)	−0.0040 (9)	0.0033 (9)	−0.0101 (9)
C30	0.0418 (12)	0.0308 (10)	0.0277 (10)	−0.0073 (9)	0.0016 (9)	−0.0023 (8)
N2	0.0435 (10)	0.0240 (8)	0.0238 (8)	−0.0070 (7)	0.0001 (7)	−0.0025 (6)
O4	0.0877 (12)	0.0262 (7)	0.0300 (8)	−0.0089 (8)	0.0107 (8)	−0.0038 (6)
O5	0.0841 (12)	0.0319 (8)	0.0335 (9)	−0.0109 (8)	0.0133 (8)	−0.0067 (7)
O6	0.0867 (12)	0.0331 (8)	0.0387 (9)	−0.0062 (8)	0.0036 (8)	−0.0147 (7)
Cl3	0.0647 (4)	0.0296 (3)	0.0360 (3)	−0.0111 (2)	0.0033 (3)	0.0010 (2)
Cl4	0.0639 (4)	0.0256 (3)	0.0524 (3)	−0.0049 (2)	0.0037 (3)	−0.0129 (2)

### Geometric parameters (Å, °)

**Table:**

C1—O1	1.241 (2)	C21—O4	1.238 (2)
C1—N1	1.339 (3)	C21—N2	1.342 (2)
C1—C2	1.481 (3)	C21—C22	1.478 (3)
C2—C3	1.336 (3)	C22—C23	1.333 (3)
C2—H2	0.93	C22—H22	0.93
C3—C4	1.489 (3)	C23—C24	1.485 (3)
C3—H3	0.93	C23—H23	0.93
C4—O3	1.213 (2)	C24—O6	1.214 (2)
C4—O2	1.298 (2)	C24—O5	1.300 (2)
C5—C10	1.388 (3)	C25—C26	1.386 (3)
C5—C6	1.394 (3)	C25—C30	1.396 (3)
C5—N1	1.415 (2)	C25—N2	1.415 (2)
C6—C7	1.381 (3)	C26—C27	1.385 (3)
C6—H6	0.93	C26—H26	0.93
C7—C8	1.387 (3)	C27—C28	1.390 (3)
C7—Cl1	1.7343 (19)	C27—Cl3	1.7271 (19)
C8—C9	1.384 (3)	C28—C29	1.377 (3)
C8—Cl2	1.7253 (19)	C28—Cl4	1.7282 (19)
C9—C10	1.374 (3)	C29—C30	1.379 (3)
C9—H9	0.93	C29—H29	0.93
C10—H10	0.93	C30—H30	0.93
N1—H1N	0.86	N2—H2N	0.86
O2—H2A	0.89 (2)	O5—H5A	0.90 (2)
			
O1—C1—N1	122.47 (18)	O4—C21—N2	122.46 (17)
O1—C1—C2	123.38 (18)	O4—C21—C22	123.11 (17)
N1—C1—C2	114.15 (18)	N2—C21—C22	114.43 (17)
C3—C2—C1	128.32 (19)	C23—C22—C21	128.12 (19)
C3—C2—H2	115.8	C23—C22—H22	115.9
C1—C2—H2	115.8	C21—C22—H22	115.9
C2—C3—C4	132.04 (19)	C22—C23—C24	132.64 (19)
C2—C3—H3	114	C22—C23—H23	113.7
C4—C3—H3	114	C24—C23—H23	113.7
O3—C4—O2	120.48 (19)	O6—C24—O5	119.99 (19)
O3—C4—C3	118.52 (18)	O6—C24—C23	118.96 (18)
O2—C4—C3	121.00 (18)	O5—C24—C23	121.05 (17)
C10—C5—C6	119.89 (18)	C26—C25—C30	119.63 (17)
C10—C5—N1	116.76 (17)	C26—C25—N2	123.62 (17)
C6—C5—N1	123.36 (18)	C30—C25—N2	116.75 (17)
C7—C6—C5	119.00 (18)	C27—C26—C25	119.21 (18)
C7—C6—H6	120.5	C27—C26—H26	120.4
C5—C6—H6	120.5	C25—C26—H26	120.4
C6—C7—C8	121.28 (18)	C26—C27—C28	121.11 (18)
C6—C7—Cl1	118.55 (15)	C26—C27—Cl3	118.51 (15)
C8—C7—Cl1	120.16 (15)	C28—C27—Cl3	120.37 (14)
C9—C8—C7	118.99 (18)	C29—C28—C27	119.39 (17)
C9—C8—Cl2	119.23 (15)	C29—C28—Cl4	119.48 (15)
C7—C8—Cl2	121.78 (15)	C27—C28—Cl4	121.13 (15)
C10—C9—C8	120.57 (19)	C28—C29—C30	120.20 (18)
C10—C9—H9	119.7	C28—C29—H29	119.9
C8—C9—H9	119.7	C30—C29—H29	119.9
C9—C10—C5	120.27 (19)	C29—C30—C25	120.46 (18)
C9—C10—H10	119.9	C29—C30—H30	119.8
C5—C10—H10	119.9	C25—C30—H30	119.8
C1—N1—C5	127.91 (17)	C21—N2—C25	128.46 (16)
C1—N1—H1N	116	C21—N2—H2N	115.8
C5—N1—H1N	116	C25—N2—H2N	115.8
C4—O2—H2A	112 (2)	C24—O5—H5A	109.4 (19)
			
O1—C1—C2—C3	7.9 (4)	O4—C21—C22—C23	1.3 (4)
N1—C1—C2—C3	−172.1 (2)	N2—C21—C22—C23	−179.0 (2)
C1—C2—C3—C4	1.2 (4)	C21—C22—C23—C24	0.0 (4)
C2—C3—C4—O3	174.2 (2)	C22—C23—C24—O6	−175.8 (2)
C2—C3—C4—O2	−6.2 (4)	C22—C23—C24—O5	3.9 (4)
C10—C5—C6—C7	0.3 (3)	C30—C25—C26—C27	−0.6 (3)
N1—C5—C6—C7	−179.75 (19)	N2—C25—C26—C27	179.24 (18)
C5—C6—C7—C8	−0.2 (3)	C25—C26—C27—C28	0.2 (3)
C5—C6—C7—Cl1	−179.91 (15)	C25—C26—C27—Cl3	179.23 (15)
C6—C7—C8—C9	−0.2 (3)	C26—C27—C28—C29	0.3 (3)
Cl1—C7—C8—C9	179.47 (15)	Cl3—C27—C28—C29	−178.67 (16)
C6—C7—C8—Cl2	−179.39 (15)	C26—C27—C28—Cl4	−178.99 (16)
Cl1—C7—C8—Cl2	0.3 (2)	Cl3—C27—C28—Cl4	2.0 (3)
C7—C8—C9—C10	0.5 (3)	C27—C28—C29—C30	−0.5 (3)
Cl2—C8—C9—C10	179.72 (16)	Cl4—C28—C29—C30	178.78 (17)
C8—C9—C10—C5	−0.4 (3)	C28—C29—C30—C25	0.2 (3)
C6—C5—C10—C9	0.0 (3)	C26—C25—C30—C29	0.3 (3)
N1—C5—C10—C9	−179.96 (19)	N2—C25—C30—C29	−179.47 (18)
O1—C1—N1—C5	−5.8 (4)	O4—C21—N2—C25	0.7 (3)
C2—C1—N1—C5	174.17 (19)	C22—C21—N2—C25	−179.03 (18)
C10—C5—N1—C1	−155.1 (2)	C26—C25—N2—C21	−1.7 (3)
C6—C5—N1—C1	24.9 (3)	C30—C25—N2—C21	178.07 (19)

### Hydrogen-bond geometry (Å, °)

**Table:**

*D*—H···*A*	*D*—H	H···*A*	*D*···*A*	*D*—H···*A*
N1—H1N···O6^i^	0.86	2.03	2.869 (2)	165
N2—H2N···O3^ii^	0.86	2.03	2.873 (2)	166
O2—H2A···O1	0.89 (2)	1.61 (2)	2.496 (2)	171 (3)
O5—H5A···O4	0.90 (2)	1.59 (2)	2.492 (2)	174 (3)

Symmetry codes: (i) −*x*+2, −*y*+1, −*z*+1; (ii) −*x*+1, −*y*+1, −*z*+2.
